# Bilateral Tibialis Anterior Muscle Herniation at the Superficial Peroneal Nerve Fascial Exit Point in a Professional Footballer: A Nerve-Sparing Primary Repair

**DOI:** 10.7759/cureus.109807

**Published:** 2026-05-28

**Authors:** Muhammed Yusuf Afacan, Okan Can Karadeniz, Furkan Özönder, Süha Ahmet Aktaş, Ali Can Baris

**Affiliations:** 1 Department of Orthopaedics and Traumatology, University of Health Sciences, Istanbul Physical Therapy and Rehabilitation Training and Research Hospital, Istanbul, TUR; 2 Department of Anatomy, Istanbul University-Cerrahpaşa, Institute of Graduate Studies, Istanbul, TUR

**Keywords:** dynamic ultrasonography, exertional leg pain, fascial defect, muscle hernia, primary fascial repair, professional athlete, superficial peroneal nerve, tibialis anterior muscle herniation

## Abstract

Tibialis anterior muscle herniation is an uncommon and often under-recognized cause of exertional anterolateral leg pain in athletes. We report the case of a young professional footballer who presented with bilateral painful anterolateral leg swellings that became prominent during activity and muscle contraction. Dynamic ultrasonography demonstrated bilateral focal fascial defects involving the tibialis anterior compartment and confirmed the diagnosis. Intraoperatively, both defects were located at the points where the superficial peroneal nerves perforated the crural fascia. Bilateral primary anatomical fascial repair was performed through small incisions with meticulous nerve preservation. At 12 weeks postoperatively, the patient returned to sport-specific training without recurrent swelling, pain, or neurological symptoms. This case emphasizes the diagnostic value of dynamic ultrasonography for intermittent exertional leg masses at a locus minoris resistentiae around neurovascular fascial exit points and highlights nerve-sparing primary repair as a practical, implant-free, cost-effective, and function-preserving option for selected athletes with small symptomatic fascial defects.

## Introduction

Muscle herniation, defined as the protrusion of the muscle belly through a defect in the superficial fascia, is a relatively rare clinical entity classified as either congenital or acquired [1]. While congenital defects are often attributed to general fascial weakness, acquired cases are predominantly secondary to direct trauma or chronic compartmental pressure associated with hypertrophy [2,3]. Consequently, these lesions are most frequently observed in the lower extremities of young, physically active individuals, such as athletes and military personnel, who are subjected to excessive strenuous leg activities [2,3].

Among lower extremity muscle herniations, the tibialis anterior muscle is the most commonly affected site due to the vulnerability of its superficial fascia [2,3]. The tibialis anterior muscle lies in the anterior compartment of the leg and functions primarily as an ankle dorsiflexor [3]. In contrast, the superficial peroneal nerve descends within the lateral compartment and becomes subcutaneous after perforating the deep crural fascia [3]. This fascial perforation site may represent a focal area of relative weakness, particularly in athletes exposed to repetitive increases in compartmental pressure. Clinically, these hernias typically present as a soft tissue swelling that becomes more prominent during muscle contraction, such as ankle dorsiflexion, or in the standing position and often reduces or disappears when the muscle is relaxed or the patient is supine [4]. Although frequently asymptomatic, symptomatic cases may present with pain, cramping, or tenderness, particularly following intense physical activity.

Due to their rarity and variable presentation, muscle hernias can be easily misdiagnosed as other soft tissue masses, including lipomas, hematomas, fibromas, or varicosities [5]. While the diagnosis can often be suspected based on physical examination, accentuated by maneuvers such as the "fencer's lunge", dynamic ultrasonography is considered the gold standard for confirmation, as it allows for the real-time visualization of the fascial defect and muscle protrusion [6,7]. Magnetic resonance imaging (MRI) may be utilized in equivocal cases to evaluate the surrounding soft tissues and rule out other pathologies [3,5]. Despite the limited number of reports concerning tibialis anterior muscle herniation, Ceyhan et al. have described bilateral involvement [8].

In this report, we present a rare case of bilateral symptomatic tibialis anterior muscle herniation in a young professional footballer, diagnosed using dynamic ultrasonography and treated with bilateral nerve-sparing primary fascial repair. Beyond documenting an uncommon bilateral presentation in a high-demand sporting context, the novelty of this case lies in the bilateral intraoperative demonstration that both fascial defects were located precisely at the superficial peroneal nerve fascial exit points. This anatomical finding supports the concept that neurovascular perforation sites may represent focal zones of fascial weakness. By integrating dynamic ultrasonographic confirmation, bilateral anatomical correlation, meticulous nerve preservation, tension-free primary repair, and successful return to sport, this case highlights the diagnostic and therapeutic relevance of a nerve-sparing anatomical approach for selected symptomatic tibialis anterior muscle herniations in high-demand athletes.

## Case presentation

A 17-year-old male professional football player presented to our outpatient clinic with complaints of palpable and painful swelling on the anterolateral aspect of both tibiae. The patient had no history of trauma or known comorbidities. He reported that the swelling became significantly more prominent during walking, running, and knee hyperflexion. While he described no pain at rest, he complained of exertional pain over the swollen areas during football activities, which hindered his athletic performance. The symptoms had been progressively worsening over the past three months. On physical examination, bilateral focal prominences were noted over the anterolateral aspect of the middle third of the legs, corresponding to the tibialis anterior compartment. The swellings were minimally apparent at rest but became more prominent during provocative maneuvers, including walking, running, knee hyperflexion, and active ankle dorsiflexion. The lesions were tender on palpation during provocation. Manual reduction was not applicable as a fixed reducible mass, because the protrusions were intermittent and exertion-dependent rather than persistently visible at rest; they subsided spontaneously after relaxation. The patient also described stabbing discomfort and intermittent tingling over the swelling sites during exertion. No persistent motor weakness or objective sensory deficit was detected at rest.

**Figure 1:**
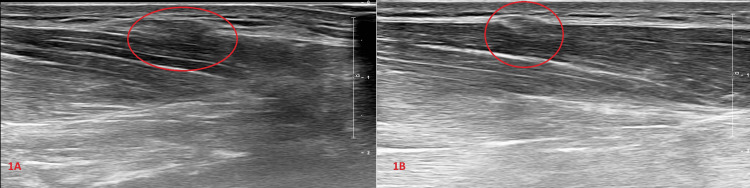
Dynamic ultrasonography of bilateral tibialis anterior muscle herniation A (right), B (left): Longitudinal ultrasonographic images showing focal fascial defects at the middle third of both crura, with protrusion of the tibialis anterior muscle through the fascia. The red circles indicate the herniation sites.

Upon clinical suspicion of tibialis anterior muscle herniation, an initial ultrasound examination was performed. The bilateral ultrasound report indicated a "loss of integrity of the tibialis anterior muscle fascia at the middle third of the crus, with a fascial defect area measuring approximately 2 cm in transverse diameter" (Figure 1A-1B). Consequently, surgical intervention was planned. Preoperatively, it was anticipated that the defects could be repaired primarily due to their small size; however, preparations were made for a tensor fasciae latae (TFL) autograft in the event of a larger-than-expected defect or inability to achieve primary closure.

**Figure 2:**
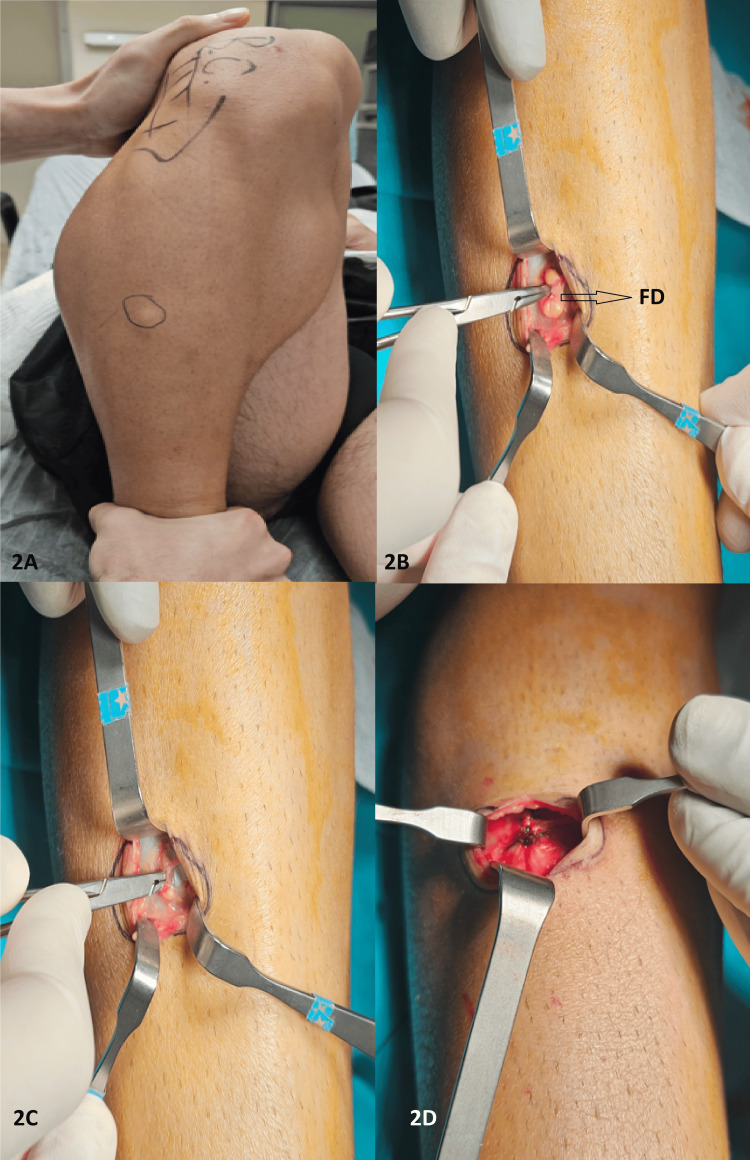
Clinical and intraoperative findings of right tibialis anterior muscle herniation (A) Preoperative marking of the palpable herniation site on the anterolateral aspect of the right leg, accentuated by knee hyperflexion. (B) Intraoperative exposure through a small incision demonstrating the fascial defect and herniated tibialis anterior muscle. (C) The herniation was reduced at the point where the superficial peroneal nerve perforates the crural fascia. (D) Primary anatomical repair of the fascial defect was performed with meticulous preservation of the superficial peroneal nerve. FD: fascial defect

**Figure 3:**
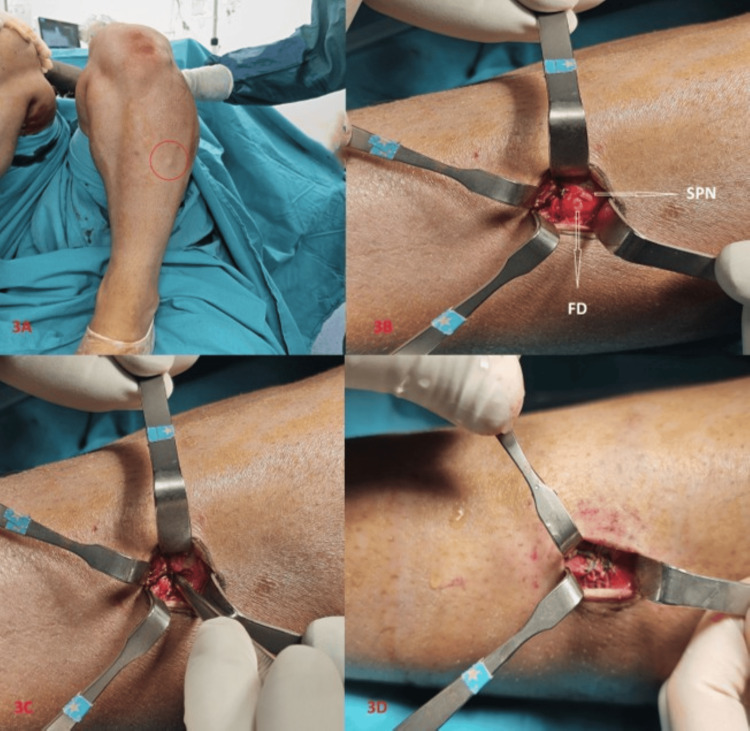
Clinical and intraoperative findings of left tibialis anterior muscle herniation (A) Preoperative marking of the palpable herniation site on the anterolateral aspect of the left leg, accentuated by knee hyperflexion. (B) Intraoperative exposure through a small incision demonstrating the fascial defect and herniated tibialis anterior muscle. (C) The herniation was reduced at the point where the superficial peroneal nerve perforates the crural fascia. (D) Primary anatomical repair of the fascial defect was performed with meticulous preservation of the superficial peroneal nerve. FD: fascial defect; SPN: superficial peroneal nerve

Under spinal anesthesia, the patient was placed in the supine position. Both knee joints were brought into hyperflexion to provoke the herniation, and the sites were marked (Figure 2A and Figure 3A). A 1 cm incision was made over the herniation on the right lower extremity, exposing the fascial tear (Figure 2B). Intraoperatively, the herniation site was identified exactly at the point where the superficial peroneal nerve perforates the fascia (Figure 2B-2C). The herniated muscle was reduced, and the fascia was repaired primarily while meticulously preserving the nerve (Figure 2D and Figure 3D). Subsequently, the procedure was repeated on the left lower extremity (Figure 3). Following a 1 cm incision over the hernia, the defect was similarly found to be located at the point where the superficial peroneal nerve becomes superficial (Figure 3B-3C). The nerve structure was preserved, the herniation was reduced, and the fascia was repaired primarily (Figure 3D).

Postoperatively, the patient reported an early reduction in pain. Sutures were removed at two weeks, and the patient was managed with a walker boot for six weeks. After the sixth postoperative week, light jogging and supervised physical therapy were initiated. At 12 weeks postoperatively, the patient was allowed to return to sport-specific training after meeting predefined clinical criteria, including complete wound healing, absence of recurrent swelling, pain, tenderness, or paresthesia during daily activities and light jogging, painless full ankle and knee range of motion, and painless resisted ankle dorsiflexion. He then gradually progressed to football-specific drills without recurrent symptoms.

## Discussion

The principal novelty of the present case is the combination of bilateral tibialis anterior muscle herniation in an athlete, dynamic ultrasonographic confirmation, bilateral intraoperative identification of the fascial defects at the superficial peroneal nerve exit points, nerve-sparing primary repair, and successful return to sport.

Our patient presented with bilateral tibialis anterior muscle herniation, a rarely described entity in the literature that can mimic soft tissue tumors, and underwent primary fascial reconstruction. These muscle herniations are predominantly observed in the lower extremities and frequently present a diagnostic challenge due to their resemblance to soft tissue lesions such as hematomas and lipomas [5,9]. While often asymptomatic and posing primarily cosmetic concerns, these herniations can become symptomatic in athletes or following trauma in otherwise healthy individuals. Dynamic ultrasonography is particularly valuable for diagnosis because it allows the real-time visualization of the fascial defect and muscle protrusion during provocative maneuvers while remaining accessible, rapid, and cost-effective [6].

Several surgical options have been described for symptomatic tibialis anterior muscle herniation, including fasciotomy, mesh repair, fascia lata autograft reconstruction, and primary fascial repair. Fasciotomy may reduce the risk of postoperative compartment-related problems but can be associated with larger scars and cosmetic concerns. Mesh repair provides reinforcement but introduces foreign material and may be less desirable in high-demand athletes [10,11]. Fascia lata autografting avoids synthetic material but requires a second surgical site and may cause donor-site morbidity [10]. In the present case, primary repair was preferred because both defects were small and could be closed without excessive tension. This approach allowed anatomical restoration without implant use or donor-site morbidity, while careful dissection enabled the preservation of the superficial peroneal nerve. However, the main limitation of this report is the relatively short follow-up period. Although the patient returned to sport-specific training at 12 weeks without recurrent swelling, pain, or neurological symptoms, longer follow-up is required to confirm the durability of the repair, the absence of recurrence, and full return to competitive play.

Our case aims to contribute to the literature regarding these controversies. Supporting the theory that muscle hernias occur at "points of fascial weakness", the herniation in our patient occurred bilaterally exactly where the superficial peroneal nerve perforates the fascia, notably without a history of direct trauma. This bilateral anatomical concordance supports the concept that neurovascular perforation sites may act as a locus minoris resistentiae or a focal zone of fascial weakness. This finding is clinically relevant because repair in this region requires careful dissection and direct awareness of the superficial peroneal nerve to avoid iatrogenic nerve injury. Furthermore, primary repair was selected due to the relatively small size of the defects. The repair was performed meticulously, ensuring that the fascia was not over-tensioned or torn.

In our patient, primary repair was selected because the defects were limited in size and could be closed without excessive tension. This approach avoided foreign material, donor-site morbidity, and larger incisions while allowing bilateral preservation of the superficial peroneal nerves. At 12 weeks postoperatively, the patient returned to sport-specific training without recurrent swelling, pain, or neurological symptoms. Therefore, this case supports nerve-sparing primary fascial repair as a simple, implant-free, cost-effective, and function-preserving treatment option for selected high-demand athletes with small symptomatic tibialis anterior fascial defects.

## Conclusions

Tibialis anterior muscle herniation should be considered in young athletes with exertional anterolateral leg pain and intermittent activity-related soft tissue swelling. Dynamic ultrasonography is useful for confirming the diagnosis by demonstrating the fascial defect and muscle protrusion in real time. In selected patients with small symptomatic defects, nerve-sparing primary anatomical repair may provide a simple, implant-free, and function-preserving treatment option that can allow safe return to sport in selected high-demand athletes.
